# Coblation Versus Diode Laser Tonsillectomy: A Comparative Study

**DOI:** 10.22038/IJORL.2021.56901.2961

**Published:** 2022-03

**Authors:** Ameer A. Alaqeedy, Raid M. Al-Ani, Rasheed Ali Rashid

**Affiliations:** 1 *Department of Surgery/Otolaryngology, College of Medicine, University of Anbar, Ramadi city, Iraq.*; 2 *Department of Surgery/Otolaryngology, College of Medicine, Tikrit University, Tikrit city, Iraq.*

**Keywords:** Coblation; Diode Laser, Tonsillectomy, Comparative Study

## Abstract

**Introduction:**

The study aimed to compare the intra-individual 2 procedures of tonsillectomy (Coblation and diode laser) concerning the operative time, the amount of blood loss, postoperative pain, and other complications.

**Materials and Methods:**

A comparative study was conducted at many Private Hospitals, Baghdad, Iraq from February 2019- February 2020. Coblation and diode laser tonsillectomy were performed on the same patient (one for each side) which was blinded to the patients or their caregivers and the surgeon who did the tonsillectomy. A double blinded randomization process of 1 to 1 of these two procedures according to the side involved was performed. Data concerning the age, gender, indications for tonsillectomy, operative time, the amount of the blood loss, post-tonsillectomy pain by VAS scores, and other complications were recorded for each patient.

**Results:**

Out of 62 participants, there were 34 (54.8%) females. The majority were from the age group ≤18 years (n = 54, 87.1%). The operative time and amount of the intraoperative blood loss were significantly lower in the Coblation than a diode laser tonsillectomy. However, the postoperative pain scores in the diode laser were a statistically significant difference less than the Coblation group at the periods 3 hours, 8 hours, 24 hours, and 7 days (P-value < 0.05).

**Conclusions:**

Coblation was better than diode laser because of shorter operative time and less intraoperative blood loss. However, the diode laser technique had lesser postoperative pain at the postoperative periods 3 hours, 8 hours, 24 hours, and 7 days than Coblation.

## Introduction

One of the most common operations in the field of otolaryngology is a tonsillectomy, which is most commonly indicated in cases of recurrent tonsillitis or obstructive upper airway due to tonsillar enlargement (1,2). Conventional dissection tonsillectomy is considered a gold standard procedure for a long period (3). Tonsillectomy is performed on both children and adult populations. Although, the procedure is common but still carries significant complications particularly postoperative pain and bleeding. There are many surgical methods of tonsillectomy used in daily practice, without consensus among otolaryngologists on the best procedure (4).

The drawback of the conventional dissection tonsillectomy is an open postoperative wound that heals by secondary intention with its sequel of postoperative pain and bleeding. Therefore, otolaryngologists use many other procedures for removal of the tonsils such as laser, electrocautery, Coblation, radio-frequency, monopolar or bipolar diathermy, and harmonic scalpel (5).

The term Coblation is derived from controlled ablation. It was first discovered by Hira V. Thapliyal and Philip E. Eggers (6). It depends on radiofrequency energy. It passes through an isotonic sodium chloride and creates a plasma field. As a result, free sodium ions are dissociated, and able to destroy the intercellular bonds and hence dissociation of the tissues. This process is achieved at a lower temperature (60-70℃) than the electrocautery (400-600℃). The cooling effect of the irrigating isotonic saline protects the surrounding tissues from the high heat (7,8). 

There are various types of lasers have been used in tonsillectomy procedure like CO2, KTP, NDYAG, and diode (9). The advantages of the diode laser are good thermal effect on the perifocal tissues with shallow depth of penetration, thus caries few side effects to the deep tissues. Therefore, it is very useful in the tonsillectomy technique because of low postoperative complications with excellent outcomes (10). There are various investigations compare the various techniques of tonsillectomy (3,5,8,11-13). The aim of these investigations was to find the best method of tonsillectomy concerning the time of the operation, intraoperative blood loss, and the complication rate. However, till now, the otolaryngologists are searching for the ideal way of tonsillectomy. As reported in the literature, two studies investigate the comparison between the Coblation and diode laser tonsillectomy. These were conducted on children, no intra-individual comparison between the two methods, and lastly, the indication for tonsillectomy recurrent tonsillitis in the study by Elbadawey et al. while, the other study the indication for tonsillectomy was not mentioned (14,15). Hence, we aimed to compare the intrapersonal Coblation and diode laser tonsillectomy in pediatrics and adult populations regarding the operative time, intraoperative blood loss, and postoperative pain and complications.

## Materials and Methods

This double-blinded randomized controlled trial was conducted at many Private Hospitals, Baghdad city, Iraq. The study covered the period of one year (February 2019 to January 2020). Patients with an age range of 5-40 years, both sexes, and with an indication of tonsillectomy due to recurrent tonsillitis or obstructive tonsillar hypertrophy of grade 4 tonsils according to the Brodsky tonsillar grading scale were enrolled in the study (16). Recurrent tonsillitis means 7 attacks of acute tonsillitis per year, or 5 per year for 2 successive years or 3 per year for 3 successive years. The exclusion criteria included patients with tonsillectomy and other procedures like adenoidectomy, and grommet insertion, previous attack of quinsy, patients who didn’t wish to participate in the study, patients with bleeding disorders, and those who lost to follow-up. Detailed information sheets on the nature of the study and the two Coblation and diode laser tonsillectomy that would be used were provided to patients or parents. The enrolled subjects were subjected to the two procedures, one for each side. The patients or parents had not told which method was used for which side, by this, the patient will be as a control for symptoms while the signs of healing were looked for which are different from patient to patient. Neither the authors nor the surgeons who did the operation were participating in the randomization process. The allocation randomization process was carried out using the simple method by adopting the odd numbers of the cases, in which the diode laser tonsillectomy was performed on the right side and the Coblation on the left side, and the opposite is true for even case numbers. This process was carried out by a person who was not involved in the research. Informed consent was taken from the patients or their parents. The current study was approved by the Ethical Approval Committee of the University Of Anbar (reference number 33, 8-4-2021). The current study was carried out according to the good clinical practice guidelines and the ethical information disclosed in the Declaration of Helsinki. We also recorded the time of surgery and amount of blood loss as the size, vascularity, state of inflammation, and adhesion would be more closely correlated in the same person than between two different patients.

***Sample size***

The calculation of the sample size was depended on similar previous tonsillectomy study (14). To reach a power of 80% and a significance level (α) of 0.05, 38 patients were required in each group. Owing to the comparison between the two methods was carried out in the same patient, and the total consecutive patient was 62. Therefore, Coblation tonsillectomy was performed on 31 right and 31 left side, with a similar number for the diode laser tonsillectomy.

***Surgical method***

All cases had general anesthesia in a supine position with a draping of the whole body exposing only the mouth, then a Boyle –Davis mouth gag was inserted with Draffin suspensors applied. The tonsil was held by tonsil holding forceps and pulled medially, by using either Coblation or diode laser handle, the anterior pillar was incised till reaching the tonsil capsule, then the tonsil dissected till reaching the inferior pole starting from the superior one. In the Coblation tonsillectomy group, we used a Coblator® II system (ENTec, Sunnyvale, California, USA) and the Evac T&A Plasma Wand (ENTec) was used at an intensity setting of 7 for dissection of the tonsils at the subcapsular plane. Bleeding points were secured using an intensity setting of 4 on coagulation mode. In the diode laser tonsillectomy group, a diode laser (980-nm wavelength with a fiber-optic delivery system; CeralasTM D25, 980 ± 30 nm, 25 W, model 003; CeramOptec, Bonn, Germany) was used. The machine was set to 5 W continuous beams for subcapsular dissection. Besides, bleeding points were secured by diode laser. The laser beam was delivered via a 0.6 mm Endostat fiber. Routine precautions for the safety of theatre personnel were followed during the use of the laser. The amount of blood loss was estimated by using standard pieces of gauze which were soaked in normal saline then squeezed then weighed by an electric scale before and after it is used to dry blood oozing from the incision or dissection. 

All procedures were performed by well expertise surgeons in the field of the diode laser and Coblation tonsillectomy. The time taken to remove each tonsil was measured by minutes and any complications that occurred during surgery were recorded. After surgery, the patients were kept in the ward, monitored for vital signs and signs of bleeding regularly (six-hourly for 24 hours) then they were discharged home. The two sides were compared for pain in the throat and healing signs at 3 hours, 8 hours, 24 hours, 7 days, and 2 weeks post-operatively. Intravenous and oral paracetamol per body weight was the analgesic used according to the standard pain control regime in all cases given 3 times per day for the first day. Thereafter, the analgesic is used according to the need of the patients. Additionally, prophylactic amoxicillin for the first 10 post-tonsillectomy periods was given for all patients. All patients were seen at 7, and 14 postoperative days in the outpatient clinic. 

Pain assessment depends on a 0-10 visual analog scale (VAS) score of pain, the mean ± SD was measured for each subject. The VAS was valid and used in the past for the assessment of postoperative pain in children and adults after tonsillectomy (17,18). 

The data were entered and analyzed using IBM SPSS version 25 for windows. The categorical variables were presented in a simple table of the frequencies and percentages. Chi-squared test was used to compare the categorical variables between the Coblation and diode laser tonsillectomy. Independent sample T-Test was used to compare the means of the variables. P-value at <0.05 considered a statistically significant difference. For the calculation of the clinical effect size, we used Paired Sample *t* Test of the two identical continuous variables to find the means of them plus SD. According to Cohen's equation, the clinical effect size = (mean 1- mean 2)/SD. If the result is 0.2 means small, 0.5 moderate, and 0.8 large effect.

## Results

Sixty-two patients fulfilled the inclusion criteria and enrolled in the study. The age range of the patients was 8-31 years with a median age was 9 years with an inter quarter range (IQR) 3. The most involved age group was ≤ 18 years (n = 54, 87.1%). Females (n=34, 54.8%) out the number of the males (n = 28, 45.2%), with a male/female ratio of 1/1.21 (Table 1). 

**Table 1:** The distribution of the 62 patients, with 62 Coblation tonsillectomy on one side and 62 diode laser on other side, according to the age group, gender, and indications for tonsillectomy

**Variable**	**Tonsillectomy procedures**		
	**Coblation N = 62**	**Diode laser N = 62**	**P-value**
Age groups (years)			
≤18 years	54 (87.1%)	54 (87.1%)	1.000
>18 years	8 (12.9%)	8 (12.9%)	
Gender			
Males	28 (45.2)	28 (45.2)	1.000
Females	34 (54.8)	34 (54.8)	
Indication for tonsillectomy			
Recurrent infection	40 (64.5)	40 (64.5)	1.000
Tonsillar enlargement	22 (35.5)	22 (35.5)	
			

Recurrent infection comprises 64.5% (n = 40) of the tonsillectomy indications Table 1. No statistically significant differences were found between the Coblation and diode laser tonsillectomy regarding the age groups, gender, and indication of tonsillectomy (P-value = 1.000). The mean operative time for Coblation and diode laser tonsillectomy was 10.39 minutes ± 2.059 and 11.61 minutes ± 2.228 respectively with a statistically significant difference between the two groups (P- value = 0.002). Besides, there was a statistically significant difference between the two groups (P- value = 0.003) regarding the amount of the mean blood loss (21.61 ml ± 3.973 for Coblation and 23.87 ml ± 4.287 diode laser tonsillectomy). The mean VAS of pain at the 3, 8, 24 hours, 7 days, and 2 weeks for the Coblation group were 8.71±0.857, 8.45±0.953, 7.24±0.824, 0.31±0.841, and 0.10±0.564 respectively, while in the diode laser group 8.10±0.918, 7.97±0.923, 6.65±0.832, 0.03±0.178, and 0.03±0.178 respectively. Besides, the mean VAS of pain was statistically significantly less in the diode laser than the Coblation group at the first 4 postoperative periods (P-value < 0.05). While there was no statistically significant difference between the 2 groups at the 2 weeks postoperative period (P-value = 0.392) Table 2. There were a moderately clinically significant (clinical effect size≥0.5) of all statistically significant differences parameters between the two methods of tonsillectomy a part from the post-operative pain at 7 days had a small clinically significant (clinical effect size = 0.4). There were no postoperative primary or secondary bleeding or other complications in all patients. At 2 weeks follow-up visit, the tonsillar fossae of all patients were completely healed without sloughing and necrotic tissues.

**Table 2:** The relationship between the type of operation and the mean operative time, mean blood loss, and mean VAS scores of the post-operative pain at different periods in 62 patients

**Variable**	**Tonsillectomy**		**P-value**	**Clinical effect size**
	**Coblation**	**Diode laser**		
Mean operative time	10.39±2.059	11.61±2.228	0.002	0.6
Mean blood loss	21.61±3.973	23.87±4.287	0.003	0.5
Post-operative pain				
3 hours	8.71±0.857	8.10±0.918	0.000	0.5
8 hours	8.45±0.953	7.97±0.923	0.005	0.5
24 hours	7.24±0.824	6.65±0.832	0.000	0.6
7 days	0.31±0.841	0.03±0.178	0.013	0.4
2 weeks	0.10±0.564	0.03±0.178	0.392	----
				

## Discussion

Despite tonsillectomy is one of the most common surgical procedures performed by an otolaryngologist through various techniques, postoperative bleeding and pain remain complications of this operation. The otolaryngologists try to practice and compare different procedures of tonsillectomy to achieve the best way regarding the shorter operative time with less blood loss, avoidance of intra and postoperative complications including mainly post-tonsillectomy hemorrhage and pain. We aimed to compare the diode laser and Coblation tonsillectomy both in children and adults. The strength of the study was the use of both procedures in the same patient (Coblation on one side and diode laser on the other side) to lessen the bias. Similar studies used 2 methods of tonsillectomy in the same patient (11,19). The study revealed that the Coblation was better than the diode laser method regarding the shorter operative time and less blood loss during the procedure. However, the diode laser was superior to the Coblation tonsillectomy concerning postoperative pain.

Although Coblation and laser tonsillectomies are relatively new techniques, if one compares them with the cold steel dissection method, they have gained popularity in the otolaryngology practice owing to the advancement in the technology of their machines. 

In Iraq, few randomized controlled trials studied the comparison between the cold steel dissection method with CO2 laser (20), Coblation and bipolar diathermy (12), and Coblation versus dissection method (21). This study was the first study in Iraq compared the diode laser and Coblation tonsillectomy in children and adults. 

 Powell et al. in 1997 studied the effect of radiofrequency on the tissue of the tongue in animal models, both in vitro and in vivo. They concluded that radiofrequency can reduce the volume of the tongue in a perfect and controlled way (22). Since that time, many otolaryngologists start the use of Coblation tonsillectomy in their daily clinical practice (5,12,19,23). The outcomes were good in studies involving a small size of patients (19,23). Moreover, many randomized controlled studies comparing the Coblation with other techniques of tonsillectomy revealed the superiority of this kind over other procedures regarding the operative time, amount of intraoperative blood loss, and postoperative pain (14,15), Basu et al. study reported that harmonic scalpel tonsillectomy is better than Coblation in the following Parameters; operative time, intraoperative blood loss volume, and postoperative bleeding (5). Accordingly, the study is considered this method as an advanced way of tonsillectomy. Other studies reported equivocal results with other procedures. 

 In a study from China by Zhou et al.(13), they compare retrospectively the Coblation (300 patients) with Coblation and tie tonsillectomy (215 patients) in the adult population regarding the operative time, blood loss during the procedure, post-tonsillectomy pain and complications, and full recovery and return to normal activities. The study concluded that the Coblation tonsillectomy alone was statistically significantly superior to Coblation with tie regarding all the above-mentioned parameters except post-tonsillectomy bleeding, Coblation with a tie was significantly less post-operative bleeding than Coblation tonsillectomy alone. 

A recent systematic review study by Ahmed and Arya (24) reviewed 15 randomized controlled trial articles that studied tonsillectomy by laser or other procedures. They reported that the majority of laser tonsillectomy was performed by CO2 laser (n=665, 60.3%), followed by potassium-titanyl-phosphate (KTP) laser (n=238, 21.6%), and the least diode laser (n=199,18.1%). While, tonsillectomies performed by other techniques were 792 (64.7%) by cold dissection, 238 (19.4%) by diathermy, and 194 (15.8%) by Coblation method. They concluded that there is an overall improvement in laser tonsillectomy in comparison with other techniques and this may be due to increased familiarity with laser surgeries and increment in the number of laser centers across the globe. However, our study revealed that the Coblation tonsillectomy was better than the diode laser regarding the operative time and blood loss volume during surgery. The better results of Coblation in comparison with diode laser in operative time and intraoperative blood loss may be attributed to the larger probe size of the Coblation handle with a wider surface area of effect concerning the diode laser handle. Similar findings were observed in previous studies (14,15). But the results of our study (significantly lower VAS scores of postoperative pain in diode laser in comparison with Coblation technique) in contrast to the above-mentioned studies concerning postoperative pain. The significantly less postoperative pain at least in the first postoperative week with diode laser may be due to the precise cut with less necrotic tissues and less damage to surrounding structures. 

A recent study by Kumar et al. (25) reported that the operative time and bleeding during surgery were significantly low for CO2 laser in comparison with the cold steel dissection tonsillectomy group. Pain score was comparable in early post-operative days but was high towards the end of the first week. Our results revealed that the pain scores were significantly less in the laser group in the first 7 days post-tonsillectomy in comparison with Coblation (P-value < 0.05). While at 2 weeks, there was no statistically significant difference (P-value = 0.392) between the two groups. This may be attributed to two causes, firstly, the laser has a desensitizing effect on the cut ends of the nerve fibers in the few postoperative days, thereafter, the pain sensation is increased. Secondly, the thermal effect of the laser needs more time to heal, in addition to the formation of a thick slough in the tonsillar area with possible late-onset of contraction and pain (26). Of note, secondary infection may occur beneath the thick slough layer with the increased chances of the development of secondary hemorrhage. Therefore, it is suggested to use a prophylactic antibiotic in the post-tonsillectomy period by many researchers to avoid such complications. 

Post-tonsillectomy bleeding (primary or secondary) is a major complication of tonsillectomy with variable incidences (0.95%-7.8%) among previous investigations (2,5,8,14,27). The difference in these incidences may be attributed to the following; type of tonsillectomy, primary or secondary bleeding, children or adult patients, and whether an antibiotic is used as prophylactic or not. Many investigations have observed different incidences of delayed post-tonsillectomy bleeding between children and adults, with more incidence in adults than children (2,8). Fortunately, our study didn’t report a case of post-tonsillectomy bleeding. This may be an incidental finding. 

The parameters in defining the improvement of tonsillectomy operation are shorter operative time, less intraoperative blood loss, low incidence of primary or secondary post-tonsillectomy bleeding, less postoperative pain, and early recovery and return to usual activities. Despite, many researchers studied these parameters among various tonsillectomy methods (12-14,19), there is still no consensus about the best method of tonsillectomy. Therefore, the following factors should be taken into consideration when choosing the tonsillectomy procedure; availability of devices and instrumentations, the experience of the surgeon, preference of the patients or parents, and the cost of the procedure. 

There were a moderately clinically significant effect (clinical effect size ≥ 0.5) of all statistically significant differences parameters between the Coblation tonsillectomy and diode laser tonsillectomy a part from the post-tonsillectomy pain at 7 days had a small clinically significant effect (clinical effect size = 0.4). Therefore, we suggest further study on a large sample size to get a large clinical effect size. 

Although the current study tried to avoid result bias by comparing the two sides of the same patient still it did not bypass the intrapersonal differences in size, fibrosis, and blood supply between the two tonsils of the same patient. This actually cannot be overcome and may have some effect on the results but the arbitrary selection of the sides may minimize this effect. The difference among surgeons efficacy in performing the tonsillectomy by diode laser and Coblation can make some bias in the cases. Even this is minimal as they were high standard consultants in the field, it is considered another limitation of the study. The Wong–Baker FACES® pain scale is a valid test for the assessment of the post-tonsillectomy pain in children and was used in previous studies (14). However, we used VAS for postoperative pain evaluation owing to the studied cases were comprised of adults and children. Anyhow, this is the third limitation of the study because the majority of our patients were children. 

Even though the time in preparing the 2 devices didn't take into consideration when calculating the operative time in the current study, it had a drawback on the patients as it prolongs the total operative time with its risk of anesthesia. Therefore, we don't advise using 2 techniques of tonsillectomy in the same patient in daily clinical practice as here we used them for research purposes.

## Conclusion

The study revealed that the Coblation tonsillectomy was better than the diode laser concerning the operative time and amount of intraoperative blood loss. However, the diode laser had significant low postoperative pain VAS scores in the first 7 days postoperatively. The current study found that there was a moderate clinical effect size for all parameters except the pain score at the 7 post-operative day (small clinical effect size). Owing to the above three mentioned limitations of the current study, the results cannot be generalized.
